# Supplementary material for: Content-rich biological network constructed by mining PubMed abstracts
Source: BMC Bioinformatics. 2004 Oct 8;5:147. doi: 10.1186/1471-2105-5-147 (PMC528731; doi:10.1186/1471-2105-5-147)
Supplement: Additional File 5 — The original Chilibot query results of the term "long-term potentiation (LTP)" and 22 other terms, limiting the latest references analyzed to the years 1990, 1995, 2000, and 2004. [file 1471-2105-5-147-S5.bz2 › chilibotAdditionalFile5/ltp2004/html/SYNAPSIN I_CREB.html]

 


 **SYNAPSIN I** and **CREB** 
  
Found 8 abstracts in PubMed,  **8 abstracts were retrieved and analyzed**.  


---

 Search Google  |
 PDF files only 
|  EDU domain only 

---

**Interactive relationship** (e.g. stimulation, inhibition, etc)

**Parallel relationship** (e.g. studied together, co-existance, homology, etc.)

- The combination of FPI and the HFS diet had more dramatic effects on the active state phosphorylated of  **synapsin I**  and  **CREB** .  Ref: 12770552 Neuroscience, 2003
- Levels of  **synapsin I**  and  **CREB**  decreased in relation to decreases in BDNF levels.  Ref: 12770552 Neuroscience, 2003
- BDNF has a strong effect on synaptic plasticity via the action of  **synapsin I**  and cAMP response element binding protein  **CREB** , therefore, we assessed changes in  **synapsin I**  and  **CREB**  in conjunction with BDNF.  Ref: 12770552 Neuroscience, 2003
- The use of a novel microbead injection method in our blocking experiments and Taqman reverse transcription polymerase reaction RT PCR for RNA quantification, have enabled us to evaluate the contribution of different pathways to the exercise induced increases in the mRNA levels of BDNF, TrkB,  **CREB** , and  **synapsin I** .  Ref: 14622908 Neuroscience, 2003
- **Synapsin I**  gene expression was not induced by either elevated intracellular cAMP concentration or by overexpression of protein kinase A, although a similar pattern of proteins, including  **CREB** , bound to the  **synapsin I**  and chromogranin B CRE in vitro.  Ref: 7529178 Eur J Biochem, 1994
- Exercise reversed the decrease in BDNF and its downstream effectors on plasticity such as  **synapsin I** , a molecule with a key role in the modulation of neurotransmitter release by BDNF, and the transcription factor cyclic AMP response element binding protein  **CREB** , important for learning and memory.  Ref: 14698750 Neuroscience, 2004
- **CREB**  and  **synapsin I**  have the ability to modify neuronal function by regulating gene transcription and affecting synaptic transmission, respectively.  Ref: 14622908 Neuroscience, 2003
- In fact, this impact of exercise is exemplified by our finding that BDNF regulates the mRNA levels of two end products important for neural function, i.e. cAMP response element binding  **CREB**  protein and  **synapsin I** .  Ref: 14622908 Neuroscience, 2003
